# Use of screens and intake of unhealthy food among children and adolescents: association with physical activity in a cross-sectional study

**DOI:** 10.1186/s40795-023-00763-4

**Published:** 2023-09-18

**Authors:** Anna Karolina Cerqueira Barros, Gilmar Mercês de Jesus, Graciete Oliveira Vieira, Lizziane Andrade Dias

**Affiliations:** State University of Feira de Santana, Feira de Santana, Bahia, Brazil

**Keywords:** Sedentary behavior, Eating Behavior, Students

## Abstract

**Background:**

The intake of unhealthy food taken on very regular basis may bring even further negative impact on health if associated with excessive time using of screen-based electronic devices.

**Objective:**

To estimate the association between the use of different types of screen-based devices and the intake of unhealthy foods amongst children and adolescents and to determine whether daily physical activity (DPA) has a moderating effect on the association.

**Methods:**

Cross-sectional study carried out with a probabilistic sample of students from second to fifth grade of public elementary schools in Feira de Santana, Bahia (n = 2,477; girls: 53.2%; age: 9.1 ± 1.38 years; BMI z-score 0.37 ± 4.19 Kg/m²). Food intake, screen use, and physical activity were assessed using an online questionnaire based on previous day recall (Web-CAAFE). Prevalence Ratios (PR) and 95% Confidence Intervals (95%CI) were estimated via multiple zero-inflated negative binomial regression, with adjustments for sex, age, and weekly frequency of school meal intake. The moderating effect of DPA was verified by inserting interaction terms with each screen-based device individually and with the daily sum of screen-based device exposure (∑ Screens).

**Results:**

The majority of students (72.2%) reported using screens. The intake of unhealthy foods was positively associated with the use of cell phones (PR = 1.21; 95%CI = 1.13–1.30), computers (PR = 1.33; 95%CI = 1.22–1.46), and video games (PR = 1.36; 95%CI = 1.22–1.52). TV use was inversely associated with intake of unhealthy foods (PR = 0.92; 95%CI = 0.87–0.99). DPA moderated the effect of video game use on intake of unhealthy foods, that is, among students with DPA ≥ 4 who used video games, the intake of unhealthy foods was 21% lower (PR = 0.79; 95%CI = 0. 65-0.97). Overall, ∑ Screen-based devices were associated with a 20% increase in intake of unhealthy foods.

**Conclusion:**

The intake of unhealthy foods was positively associated with the use of cell phones, computers, and video games. In addition, a frequency of four or more DPA attenuated the effect of video game exposure on intake of unhealthy foods. Upcoming investigations on the use of screen-based devices and the intake of unhealthy foods among schoolchildren should consider the exposure to different types of screen-based devices, as well as the influence of DPA.

**Supplementary Information:**

The online version contains supplementary material available at 10.1186/s40795-023-00763-4.

## Introduction

Through the latest decades, the increase in the intake of processed foods has been observed worldwide, especially in middle-income countries, due to the influence of the activity of transnational food manufacturing companies and fast food service corporations [1]. Ultra-processed foods are hyper-palatable, have low nutritional quality, high caloric density, excess salts and sugar [2, 3], such as fast food (fast or semi-ready snacks with a high content of saturated fats), sweets (packaged, with a high content of sugar with artificial colors), and sugary drinks (packaged with high durability and amount of sugar) [2]. Therefore, scientists and health authorities have become increasingly concerned about the association between the intake of these foods (unhealthy foods) [4] and negative health outcomes among children and adolescents, such as weight gain [5], obesity [1], dyslipidemia [6], and food allergies [7].

A high frequency of unhealthy foods intake may have further negative impact on health if associated with excessive time of sedentary behaviors (SB) [8, 9], which are those ones experienced during the awake state, found in the actions of sitting, reclining and lying, when there is low energy expenditure (≤ 1.5 metabolic equivalents) [10]. These characteristics are generally present when using screens (TV, computer, cell phone, tablet, or video game) [8]. Excessive use of screens is also associated with lower intake of fruits and vegetables among children and adolescents, a finding observed in both cross-sectional [11] and longitudinal studies [2, 12].

Some evidence has shown that low-quality dietary patterns identified in adolescence tend to persist into adulthood [5, 13, 14], with slight signs of improvement over the years among individuals with a lower frequency of TV use [14]. Although the recreational use of screens is a part of the daily activities of children and adolescents, reducing the excess of these behaviors is one of the current strategic objectives of the Global Action Plan for Sustainable Development 2030 of the World Health Organization [15], which could also have an effect on reducing unhealthy foods intake.

Based on these aspects, studies aiming to monitor the use of screens and food intake are important, as a way of providing data for intervention studies and strategies in order to prevent unhealthy food intake. Despite this, little is known about this matter among Brazilian children aged between 7 and 12, since national surveys on the health of schoolchildren, such as the National Survey of School Health (PeNSE) [9, 16] and the Study of Cardiovascular Risks in Adolescents (ERICA) [17] only cover adolescents aged from12 to 17 years.

In addition, as far as we were able to investigate, studies based on survey data such as the PeNSE [9, 16] and ERICA [17] did not analyze the effect of physical activity (PA) as a potential moderator of the association between screen use and unhealthy foods intake. PA is a behavior that can occur simultaneously with sedentary behavior, including the use of screens [10], and that can have an effect on hunger and satiety, and stimulate the search for food [18], regardless of whether it could be considered healthy or not [19].

Moreover, previous studies did not analyze the use of smartphones, which may underestimate the results found, as the use of cell phones by children aged 10 or older has increased since 2016 [20]. Thus, the primary objective of the current study is to estimate the association between the use of different types of screen-based devices (TV, cell phone, computer, and video game) and the intake of unhealthy foods among children and adolescents from public schools. The secondary objective is to determine whether daily physical activity (DPA) performs a moderating effect on the association.

## Methods

### Design

Cross-sectional study carried out with a probabilistic sample of students from the second to the fifth year of Elementary School in Feira de Santana, Bahia, a large city in the Northeast region of Brazil (population contingent of 624,107 inhabitants). The municipality has the fifth highest Municipal Human Development Index (MHDI = 0.712) in Bahia, given the growth through the last two decades in education, longevity, and income. It has a schooling rate of 6 to 14 years of 97.4% and an infant mortality rate of 13.85 per 1,000 live births [21].

### Sample calculation

The sample size was defined based on the following parameters: population of 15,920 students enrolled in the education network in 2019, according to data from the Municipal Department of Education; expected prevalence of the outcome of 50%; margin of sampling error of plus or minus 3% points; design effect (deff) of 2; and 95% Confidence Interval. With these parameters, the required number of participants was 2,000. A further 20% were added to compensate for losses and, thus, giving a target sample of 2,400 individuals.

The cluster sampling process was carried out in three stages: (I) all schools in the municipal network were stratified according to the 11 geographic and administrative centers of the Department of Education (clusters); (II) one school from each center was randomly drawn; (III) all classrooms from 2nd to 5th grade at each school were selected (159 classrooms), and all subjects belonging to the selected classrooms were invited to join the study.

### Participants

Children (7–9 years) and adolescents (10–12 years) from public schools, in the morning or afternoon periods, located in urban areas took part in the study. Data were collected between March and October 2019, covering only weekdays (Tuesday to Friday), and included all students with regular attendance in school, representing the 11 geographic regions where the municipal public schools were distributed. Children and adolescents with intellectual disabilities and outside the age group between seven and 12 years old participated in the study, but were excluded from the statistical analyses. The study followed the ethical standards set out in CNS Resolution No. 466/2012. All and any participants under 16 years were authorized in writing by their parents or guardians and signed an informed consent form in the declarations (ethics approval and consent to participate) section. The study protocol was approved by the Research Ethics Committee of the *Universidade Estadual de Feira de Santana* (Opinion no. 3,116,495). All methods were performed in accordance with the relevant guidelines and regulations by including a statement in the declarations.

### Variables

The exposure (use of screen-based devices), outcome (intake of unhealthy foods), and Daily Physical Activity were collected through the application of the Food Consumption and Physical Activity of Schoolchildren (Web-CAAFE) questionnaire, an internet-based monitoring instrument based on the previous day recall, and previously validated in two Brazilian cities, with consistent evidence of accuracy and reliability [22–24] available at: https://caafe.ufsc.br/portal/9/detalhes.

The instrument has an animated avatar that aids in filling it out. Regarding food intake, the participants were asked about foods and drinks consumed in the six meals/refreshments of the previous day (breakfast, morning snack, lunch, afternoon snack, dinner, and evening snack) and were able to select a list up to 30 items, from 300 stored in the system database, which they ingested in each of the meals, covering the food offered by the school: rice, vegetables (carrots, squash and broccoli), leafy greens, vegetable soup, beans, cassava flour, pasta, meat/chicken, eggs (fried, boiled or omelet), fish/seafood, corn/potato, bread /biscuits, typical fruits from all over Brazil, porridge, cheese, milk and coffee, milk and yogurt, noodles, french fries, sausages/processed foods (sausage, bologna, ham), sandwich cookies, chocolate milk, soft drinks, sweets (candy, confectioned cakes, chocolate, and ice cream), packaged snacks, fast food (pizza/hamburger/hot dog, pastel), and juice (natural and artificial).

The analyzed outcome included unhealthy foods [4]: noodles, french fries, sausages/processed foods, sandwich cookies, chocolate milk, juice (natural and artificial), soft drinks, sweets, packaged snacks and fast food. Natural juice was considered unhealthy because it is not easily distinguished by children from artificial juice when served outside of commercial packaging. In addition, the recommendations for fluid intake point to the consumption of water, predominantly, and the water included in food [25].

With regard to sedentary behaviors and physical activities, participants were asked about activities performed the previous day (morning, afternoon, and evening) and chose from a list of up to 32 icons, out of a total of 50 stored in the system, among 27 types of PA, one SB in a sitting position (academic activities: studying, drawing, painting, doing homework), and four screens (TV, video game, computer, and cell phone).

Participants completed the Web-CAAFE at the school site, in a room with laptops and headphones provided by the research team, after having been instructed about how the software works and how to complete the questionnaire, through verbal instruction aided by banners. Students were instructed not to interact during the task and the research team provided assistance when requested, without inducing responses.

The other variables studied were: age, sex, economic class, weekly frequency of school-offered meal intake, and anthropometric measurements of weight and height for the calculation of the Body Mass Index (BMI) and classification of nutritional status, according to the International Obesity Task Force reference curves [26]. Anthropometric measurements of body weight and height of the students were measured by trained researchers, following recommended standardization [27]. Weight was measured using a digital scale with a precision of 100 g, AVANUTRI brand. Height was measured using a portable, detachable stadiometer with a square platform, Seca® brand, with a maximum height of 205 cm and graduations every 1 mm. The students were barefoot, wearing their school uniform, and without any head adornments during the measurements.

Parents provided the socioeconomic status data. The socioeconomic status of the participants was defined based on the analysis of possession of items, the education of the head of the family, and access to public services, according to the Brazilian Economic Classification Criteria, of the Brazilian Association of Research Companies (ABEP) [28]. The socioeconomic level was classified into classes A (R$ 22,749.24), B-C (R$ 1,894.95 to R$ 10,788.56), and D-E (R$ 862.41), referring to the average household income in *Reais* (R$).

### Data processing and analysis

Descriptive statistics were used to present the study variables, such as proportions. Categorical variables were compared using Pearson’s chi-square test (χ²). The daily frequency of unhealthy foods intake (outcome in counting scale) was obtained by the sum, at the individual level, of all the items reported in the six meals, ranging from zero, when the participant did not report consuming any of the items presented on the screens, to 180, when all items were selected. The use of TV, cell phone, computer, and video game were categorized as exposed, when the daily frequency of reports was greater than or equal to one (report in at least one period of the day), and not exposed, when the frequency was zero (no report).

The associations between each screen-based device and the intake of unhealthy foods were evaluated by multiple zero-inflated negative binomial regression, with the other screens as adjustment factors. Every daily TV, computer, cell phone, and video game reports were added together (∑ Screens: 0 = no screens reported; 1 = report of one or more screens) to analyze the combined effect of exposure to screen-based device on unhealthy foods intake. The zero-inflated negative binomial distribution is suitable for adjusting count data susceptible to overdispersion, and showed higher linearity in the comparison between observed and predicted values of the outcome (results not shown). The variance estimator was robust and zero inflation was assumed to be constant across factors. Statistical significance was evaluated using p < 0.05.

Statistical modeling also included the following variables as adjustment factors that influence exposure and outcome variables [29, 30]: sex, age, and weekly frequency of school-offered meal intake. Daily Physical Activity (DPA) frequency (rated as 0 = no PA reported on the previous day; 1 to 3 PA; and ≥ 4 PA) was included in the modeling to ascertain their moderating effect on the association between screens and unhealthy foods intake, through the creation of interaction terms with TV, cell phone, computer, video game, and ∑ Screens. DPA were obtained by summing all reports in the morning, afternoon, and night. For example, if a participant reported riding a bike in the morning period, play catch in the afternoon, and playing with a dog in the evening, then their sum was 3 (DPA = 3). Significant interactions at the critical level of p < 0.05 were described.

## Results

Of the 4,169 students eligible for the study, 10.8% were not attending school during the data collection visits, 1.25% had transferred, 3% refused to participate, and 18.1% did not receive authorization from their parents/guardians. Among those who effectively participated, 4.3% were absent on data collection days, and 0.3% drop out. There were only two losses, of students who did not complete the Web-CAAFE. Thus, the sample obtained was 2,654 schoolchildren.

**Fig. 1:**
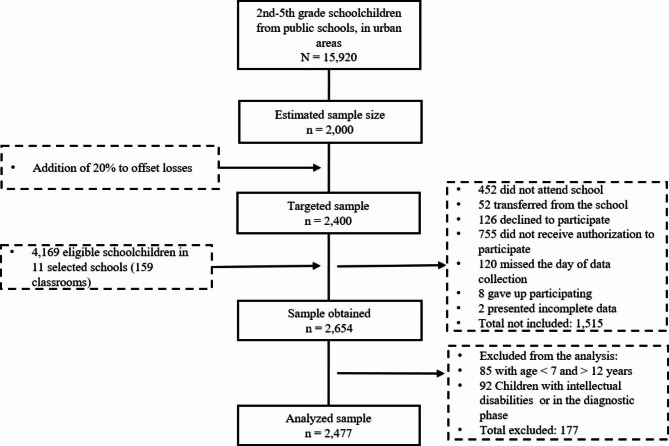
Study flowchart

Based on the exclusion criteria, data from 177 students were not analyzed, because they were outside the age group (n = 85) or because they had intellectual disabilities with a defined diagnosis (n = 24) or in the definition phase (n = 68). Thus, the analytical sample consisted of 2,477 individuals (Fig. 1). There were no differences in characteristics of excluded individuals regarding to age, weight status, weekly frequency of intake of food offered at school, or daily physical activities. However, there were differences regarding to sex and economic class (Table S1).

**Fig. 2:**
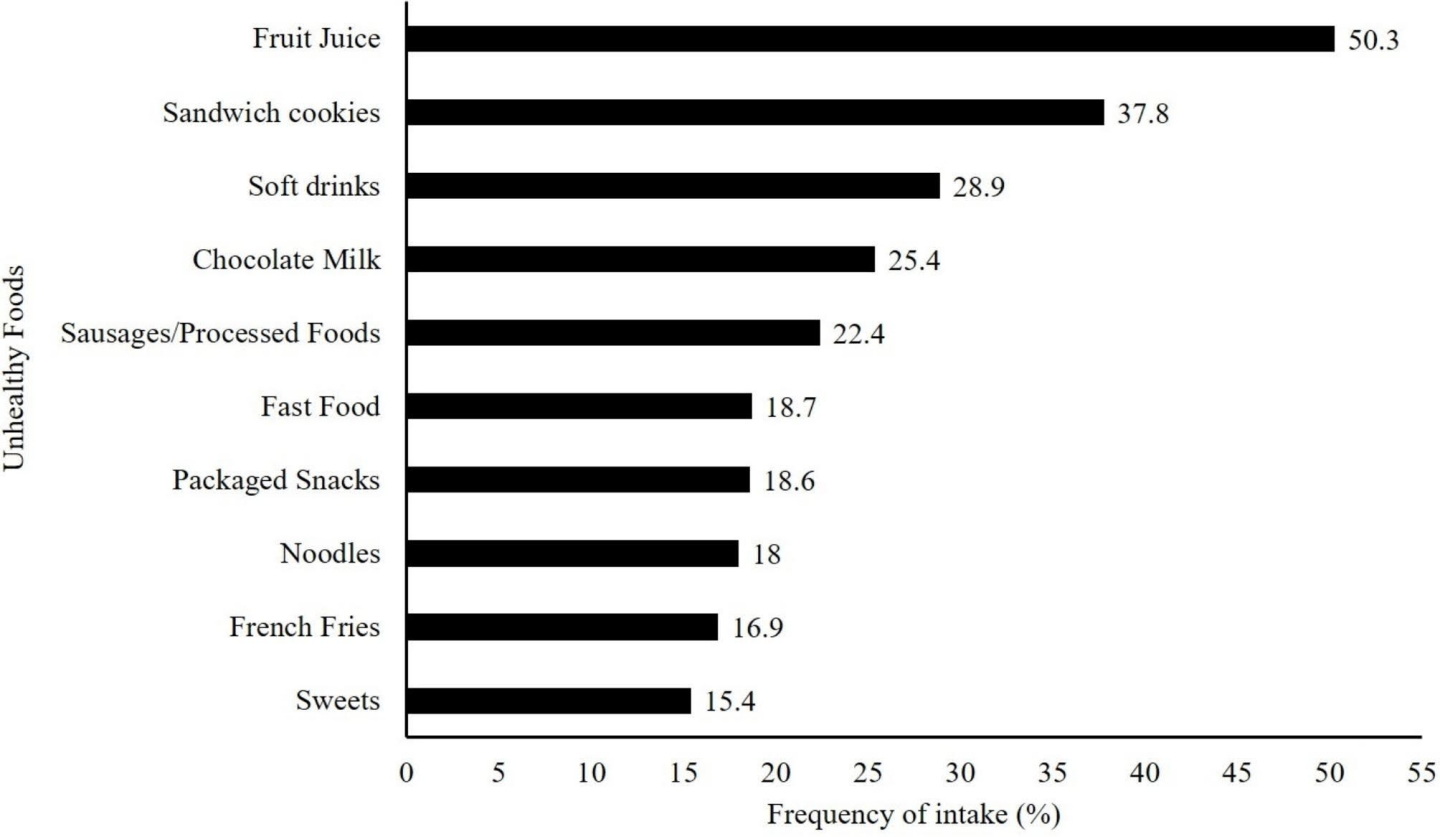
Reported unhealthy foods

Table 1 presents the characteristics of the participants. Most of the sample consisted of girls, students aged 7 to 9 years, and belonging to economic classes B-C. Overweight children and adolescents (overweight + obesity) represented 19.9% of the sample. More than half of the sample reported consuming school-offered meals at least four times a week. The majority of students reported practicing 1 to 3 PA per day and 1/3 of the sample reported more than 4 PA. Most students (72.2%) reported using screens on the previous day, with cell phones and TV being the most commonly mentioned screens used. The most widely consumed unhealthy foods were fruit juice (natural and artificial), sandwich cookies, and soft drinks (Fig. 2).

**Table 1:** Sample characteristics

Sample characteristics	n (%)	95%CI
Sex		
Girls	1,317 (53.2)	51.2–55.1
Boys	1,160 (46.8)	44.9–48.8
Age		
7–9 years	1,505 (60.8)	58.8–62.3
10–12 years	972 (39.2)	37.3–41.2
Socioeconomic class (n = 972)		
A	10 (1.1)	0.5–1.9
B-C	640 (65.8)	62.8–68.8
D-E	322 (33.1)	30.2–36.2
Weight status^a^		
Low weight/Normal Weight	1,984 (80.1)	78.5–81.6
Overweight (not obese)	333 (13.4)	12.2–14.8
Obese	160 (6.5)	5.6–7.5
Weekly frequency of School-offered food intake		
Does not ingest	203 (8.8)	7.6–10.0
1 day/week	385 (16.6)	15.2–18.3
2 days/week	253 (10.9)	9.9–12.6
3 days/week	229 (9.9)	8.8–11.3
4 days/week	477 (20.6)	18.2–22.2
Every day	770 (33.2)	31.0–35.0
Daily physical activities		
Not reported	235 (9.5)	8.4–10.7
1 to 3/day	1,415 (57.1)	55.2–59.1
≥ 4/day	827 (33.4)	31.6–35.3
Use of screens **		
Cell phone	1,215 (49.1)	47.1–51.0
TV	1,157 (46.7)	44.8–48.9
Computer	284 (11.5)	10.3–12.8
Video game	241 (9.7)	8.6–11.0
∑ Screens	1,789 (72.2)	70.4–73.9

^a^ According to IOTF reference

** Percentages refer to reports of one or more episodes per day.

The socioeconomic status assessment questionnaire had a low response rate and missing data ranged from 35 to 40.3% between items. Thus, the economic level of only 949 participants was classified and for this reason was not included as a covariate in the statistical modeling.

In the crude analysis, cell phone, computer, video game, and the ∑ screen-based device exposure were positively associated with unhealthy foods intake. On the other hand, In the adjusted analysis, these factors also remained positively associated with it, however TV use showed an inverse and significant association. Physical activity modified the effect of video game use on unhealthy foods intake and among students who reported using video games and simultaneously exhibited DPA ≥ 4, unhealthy foods intake was higher. However, among students exposed to video game and with no DPA the intake of unhealthy foods was 21% lower. There was no significant interaction of DPA with ∑ Screens (Table 2).

**Table 2:** Association measures and confidence intervals between intake of unhealthy foods and use of screen-based devices

Variable	Crude analysis		Adjusted analysis*	
	PR (95%CI)	p value	PR (95%CI)	p value
TV	1.02 (0.95–1.09)	0.534	0.92 (0.87–0.99)	0.018
Cell phone	1.31 (1.23–1.40)	0.000	1.21 (1.13–1.30)	0.000
Computer	1.60 (1.46–1.74)	0.000	1.33 (1.22–1.46)	0.000
Video game	1.49 (1.35–1.64)	0.000	1.36 (1.22–1.52)	0.000
∑ Screens	1.28 (1.17–1.39)	0.000	1.20 (1.10–1.31)	0.000
Sex (Girls)			1.22 (1.05–1.20)	0.001
Age (10–12 years)			1.08 (1.01–1.15)	0.032
School meals intake/week			0.98 (0.97–1.01)	0.129
DPA				
1–3/day			1.15 (0.98–1.34)	0.088
≥ 4/day			1.71 (1.46–1.99)	0.000
Video game *x* DPA ≥ 4^a^			1.24 (1.06–1.44)	0.007
Video game *x* DPA = 0^b^			0.79 (0.65–0.97)	0.022

*PR and 95%CI estimated with adjustment for sex, age, and weekly frequency of intake of school meals. ^a^Interaction term between Video game and Daily Physical Activity ≥ 4 physical activities reported/day.^b^Interaction term between Video game and Daily Physical Activity = no physical activities reported/day.

## Discussion

The current study investigated the association between the use of different screen-based devices and unhealthy foods intake among students aged from 7 to 12 years in public schools. Our findings revealed that exposure to cell phone, computer, and video game use was associated with higher unhealthy foods intake. On the other hand, although TV was the second most reported screen-based device by the students in the sample, its association with unhealthy foods intake was inverse. In general, students exposed to the use of any screen presented, on average, 20% higher unhealthy foods intake.

The association between watching TV and unhealthy food intake has been extensively researched and the available evidence shows that there is a strong influence of this habit on inadequate diet [11]. This influence is often attributed to the effect of advertising on TV programming that encourages the consumption of processed foods [31]. However, the findings of the present research showed lower intake of unhealthy foods among students with the habit of watching TV.

Data from the National Household Sample Survey (PNAD) on internet access, TV, and cell phone ownership amongst people over 10 years old showed that, in 2019, cell phones were the most used screens to access information and entertainment (98.6%), computer (46.2%), TV (31.9%), and tablet (10.9%) [20]. Despite the fact that 81.4% of households in the Northeast region have a TV [20], due to restrictions on advertisements for processed foods aimed at children and adolescents [32], open TV programming has changed and has become unattractive to this audience. Our findings, therefore, may be reflecting the impacts of these restrictions and the consequent change in the behavior of Brazilian children and adolescents regarding the recreational use of screens.

Video games were the type of screen-based device with the lowest frequency of use in the present research, but its effect on unhealthy food intake had the greatest magnitude. Similar results were found in cross-sectional data from European adolescents, in which a longer weekly time of video game use also led to higher odds of intake of salty snacks (only among boys) and sugary drinks [33]. A similar association was observed among Australian adolescents (12 to 18 years old), where those who played e-games were 62% more likely to consume sugary drinks regularly than those who did not, regardless of the use of other screens [34].

Additionally, video games can also influence the choice of snacks to be consumed after the games, as demonstrated in an experimental study with Australian children (7 to 12 years old) exposed to different types of advertising that promoted an unfamiliar confectionery brand in the digital games [35]. In that study, children in the group where advertising was in the design of digital games chose the product linked to the test brand more often compared to children in the other two exposure groups which were exposed to brand placement outside the game [35]. It is possible that the effect of advertising on unhealthy food intake, now restricted to TV [36], but more widely explored in other more attractive screens for children and adolescents, may explain our findings [36].

In the current study, daily physical activity, a behavior positively associated with health, was positively associated with unhealthy foods intake, although it attenuated the effect of video game use on unhealthy foods intake.

Physical activity exerts a complex influence on food intake, which can be explained, in general, by the compensatory mechanism caused by the increase in global energy expenditure among those individuals with higher levels or daily frequency of PA, which, in turn, increases food intake [18]. It should be noted that the pattern of PA in children is characterized as erratic, sporadic, and intense, with brief episodes followed by rest breaks and with rare interest in time-consuming activities [37]. In addition, hyperpalatable foods (sweet or salty) make up children’s food preferences [19]. Thus, the effect of PA accumulated throughout a child’s day, which generates a higher demand for food [18], regardless of type, can induce the consumption of unhealthy foods, if they are available [19].

On the other hand, the regular practice of PA, through participation in organized sports, can influence not only healthy food choices among adolescents, but is also associated with lower chances of them becoming involved in other unhealthy lifestyle habits [38].

Comparisons between our findings with the national and international literature should be performed with caution, due to methodological differences between the studies, with regard to groupings by age group, different ways of evaluating the foods consumed, and the methods of evaluation of exposure and outcome. In addition, the present research also contains some limitations that must be reported. First, as this is a cross-sectional study, the causal relationship cannot be estimated. In addition, questionnaires based on the 24-hour recall are susceptible to social desirability biases and can generate difficulties in understanding the data that may not reflect the usual investigated behaviors [39]. However, these questionnaires are less affected by recall bias, compared to instruments for reporting weekly habits. There were some differences between excluded individuals and the analytical sample regarding to sex and socioeconomic class that limit the generalization of the results.

In addition, and attenuating possible weaknesses of the questionnaires used, the validation studies of the Web-CAAFE revealed adequate validity of the instrument, given the low percentages of intrusion and omission of food and beverages [22, 24], which reduces the effect of these biases on the measure of food intake.

It is important to highlight that statistically significant differences were found between included and non-included subjects in the final analyses, regarding to sex and socioeconomic status. However, it is not indicating a selection bias. The possibility of selection bias is a matter of concern in epidemiological studies. However, in our study, 177 subjects were excluded from the analysis according to inclusion/exclusion criteria. Keeping the subjects with intellectual disabilities in the analytical sample could affect the variability of outcome and/or exposure variables. Likewise, subjects out of the age range of the study (> 12 years) present behaviors such as physical activity, exposure to screen-based devices, and food consumption different from 7 to 12 years old children.

As relevant points of the current study, in addition to the 24-hour recall with the inclusion of school meals among the foods consumed and the use of Web-CAAFE, a previously validated instrument, we highlight: research carried out with a representative sample of students from 2nd to 5th year of the municipal public schools of Feira de Santana; evaluation segmented by screens and with the inclusion of the cell phone; use of unusual analysis strategies in the national scientific literature; progress in adding analysis of interactions between screens with PA; in addition to the advantage of carrying out the research in the school environment, a suitable place to disseminate the results found and for educational interventions in nutritional food health.

## Conclusion

The results found showed that exposure to screen-based devices was associated with higher intake of unhealthy foods. Considering the different types of screen-based devices analyzed, students exposed to cell phones, video games, and computers showed a positive association with unhealthy food intake, but exposure to TV was negatively associated with it. In addition, a daily frequency of four or more PA attenuated the effect of video game exposure on unhealthy foods intake, but was not able to nullify it. The study findings demonstrated that investigating the association between exposure to different types of screen-based devices and unhealthy food intake can be a useful option in epidemiological research and that daily physical activity influences this association.

### Electronic supplementary material

Below is the link to the electronic supplementary material.

Supplementary Material 1

## Data Availability

The datasets generated and/or analyzed during the current study are not publicly available due to miss authorization of the Research Ethics Council but are available from the corresponding author upon reasonable request.

## List of abbreviations

DPA: Daily Physical Activity
BMI: Body Mass Index
PR: Prevalence Ratios
CI: Confidence Intervals
PeNSE: *Pesquisa Nacional de Saúde do Escolar* (National Survey of School Health)
ERICA: *Estudo de Riscos Cardiovasculares em Adolescentes* (Study of Cardiovascular Risks in Adolescents)
PA: Physical Activity
ABEP: *Associação Brasileira de Empresas de Pesquisa* (Brazilian Association of Research Companies)
MHDI: Municipal Human Development Index
DEFF: Design Effect
CNS: *Conselho Nacional de Saúde* (National Health Council)
IOTF: International Obesity Task Force
PNAD: *Pesquisa Nacional por Amostra de Domicílios* (National Household Sample Survey)
