# Supplementary material for: Use of screens and intake of unhealthy food among children and adolescents: association with physical activity in a cross-sectional study
Source: BMC Nutr. 2023 Sep 18;9:104. doi: 10.1186/s40795-023-00763-4 (PMC10507888; doi:10.1186/s40795-023-00763-4)
Supplement: Supplementary file 1 — Supplementary Material 1 [file 40795_2023_763_MOESM1_ESM.docx]

| Table S1 - Characteristics of excluded subjects. | | | |
| --- | --- | --- | --- |
| Sample characteristics | Participants n (%) | Excluded  n (%) | p value |
| Sex |  |  |  |
| Girls | 1,317 (53.2) | 62 (35.03) | 0.000 |
| Boys | 1,160 (46.8) | 115 (64.97) |  |
| Age |  |  |  |
| 7-9 years | 1,505 (60.8) | 52 (58.43) | 0.658 |
| 10-12 years | 972 (39.2) | 37 (41.57) |  |
| Socioeconomic class (n=972) |  |  |  |
| A | 10 (1.1) | - | - |
| B-C | 640 (65.8) | 35(61.40) | 0.000 |
| D-E | 322 (33.1) | 22 (38.60) | 0.000 |
| Weight statusª |  |  |  |
| Low weight/Normal Weight | 1,984 (80.1) |  |  |
| Overweight (not obese) | 333 (13.4) | 23 (13.14) | 0.910 |
| Obese | 160 (6.5) | 12 (6.86) | 0.836 |
| Weekly frequency of intake of food offered at school |  |  |  |
| Does not consume | 203 (8.8) | 15(9.49) | 0.669 |
| 1 day/week | 385 (16.6) | 25 (15.82) |  |
| 2 days/week | 253 (10.9) | 12 (7.59) |  |
| 3 days/week | 229 (9.9) | 13 (8.23) |  |
| 4 days/week | 477 (20.6) | 35 (22.15) |  |
| Every day | 770 (33.2) | 58 (36.71) |  |
| Daily physical activities |  |  |  |
| Not reported | 235 (9.5) | 22 (12.43) | 0.421 |
| 1 to 3/day | 1,415 (57.1) | 95 (53.67) |  |
| ≥ 4/day | 827 (33.4) | 60 (33.90) |  |
| Use of screens ** |  |  |  |
| Cell phone | 1,215 (49.1) | 82 (46.33) | 0.484 |
| TV | 1,157 (46.7) | 80 (45.20) | 0.697 |
| Computer | 284 (11.5) | 28 (15.82) | 0.082 |
| Video game | 241 (9.7) | 20 (11.30) | 0.498 |
| ∑ Screens | 1,789 (72.2) |  |  |
| ª According to IOTF reference | |  |  |
| ** Percentages refer to reports of one or more episodes per day. | |  |  |
